# Association of nonalcoholic fatty liver disease and venous thromboembolic disease in healthy adults in Korea: a nationwide study

**DOI:** 10.1038/s41598-023-42963-9

**Published:** 2023-09-26

**Authors:** Chang-Yeon Kim, Namkyun Kim, Jae-Hyung Roh

**Affiliations:** 1 Department of Internal Medicine, School of Medicine, Daegu Catholic University Medical Center, Daegu, Republic of Korea; 2 Department of Internal Medicine, School of Medicine, Kyungpook National University, Kyungpook National University Hospital, Daegu, Republic of Korea; 3 Department of Cardiology in Internal Medicine, School of Medicine, Chungnam National University, Chungnam National University Sejong Hospital, Sejong, Republic of Korea

**Keywords:** Endocrine system and metabolic diseases, Thrombosis

## Abstract

Nonalcoholic fatty liver disease (NAFLD) can lead to a prothrombotic state, which significantly burdens public healthcare systems. This study investigated the relationship between NAFLD and the incidence of venous thromboembolism (VTE) in Korea using National Health Insurance Service-National Sample Cohort 2.0 data. A population-based retrospective cohort analysis was conducted on 472,212 healthy individuals who underwent national health check-ups in Korea from 2009 to 2014. NAFLD was defined using the fatty liver index (FLI). Multivariate Cox proportional hazards regression models were used to analyze the association between FLI and VTE. Individuals were categorized into four quartiles according to FLI values (first quartile [Q1], 0–5.7; second quartile [Q2], 5.8–15.3; third quartile [Q3], 15.4–37.2; and fourth quartile [Q4], > 37.2). The incidence of VTE tended to increase with increasing FLI values (Q1, 598 [0.5%]; Q2, 1,033 [0.9%]; Q3, 1,443 [1.2%]; and Q4, 1,425 [1.2%]). In the age- and sex-adjusted multivariate model, the hazard ratio (HR) (95% confidence interval [CI]) was 1.47 (1.33‒1.62) for Q4 compared with Q1. After adjusting for clinical variables with *P* < 0.1 in the univariate analyses, the HR (95% CI) was 1.45 (1.30‒1.62) for Q4 compared with Q1. FLI was related to VTE risk, as confirmed after adjusting for other risk factors.

## Introduction

Nonalcoholic fatty liver disease (NAFLD) refers to a broad spectrum of liver diseases ranging from steatosis to nonalcoholic steatohepatitis (NASH) and cirrhosis. It has become increasingly prevalent worldwide, placing a significant burden on public healthcare systems^1^. The fatty accumulation in NAFLD is not only confined to the liver. It is a multisystem disease with possible extrahepatic manifestations, including colorectal cancer, cardiovascular disease (CVD), type 2 diabetes, metabolic syndrome, chronic kidney disease, polycystic ovary syndrome, and obstructive sleep apnea^2^.

Aside from NAFLD, venous thromboembolism (VTE) is also an emerging health problem and a leading cause of death worldwide^3, 4^. It is caused by various risk factors, including immobility, trauma, cancer, age, genetic or acquired thrombophilia, oral contraceptives, and obesity^5^. The development of VTE is related to venous stasis, hypercoagulability, and endothelial cell damage, which are known as Virchow’s triad.

Studies found that NAFLD is a “thrombophilic state”^6–8^ and CVD, to some extent attributable to arterial thrombosis, are the leading causes of death in patients with NAFLD^1, 9^. Several studies have investigated the relationship between advanced liver disease and increased risk of VTE^10–12^. However, only few small sample-sized studies revealed the association between fatty liver and VTE risk and their underlying mechanisms^13–15^, while only one observational study had a sufficient sample size^11^. Therefore, the present study aimed to assess the relationship between NAFLD and the incidence of VTE among healthy Korean adults.

## Results

### Baseline characteristics of participants

**Figure 1:**
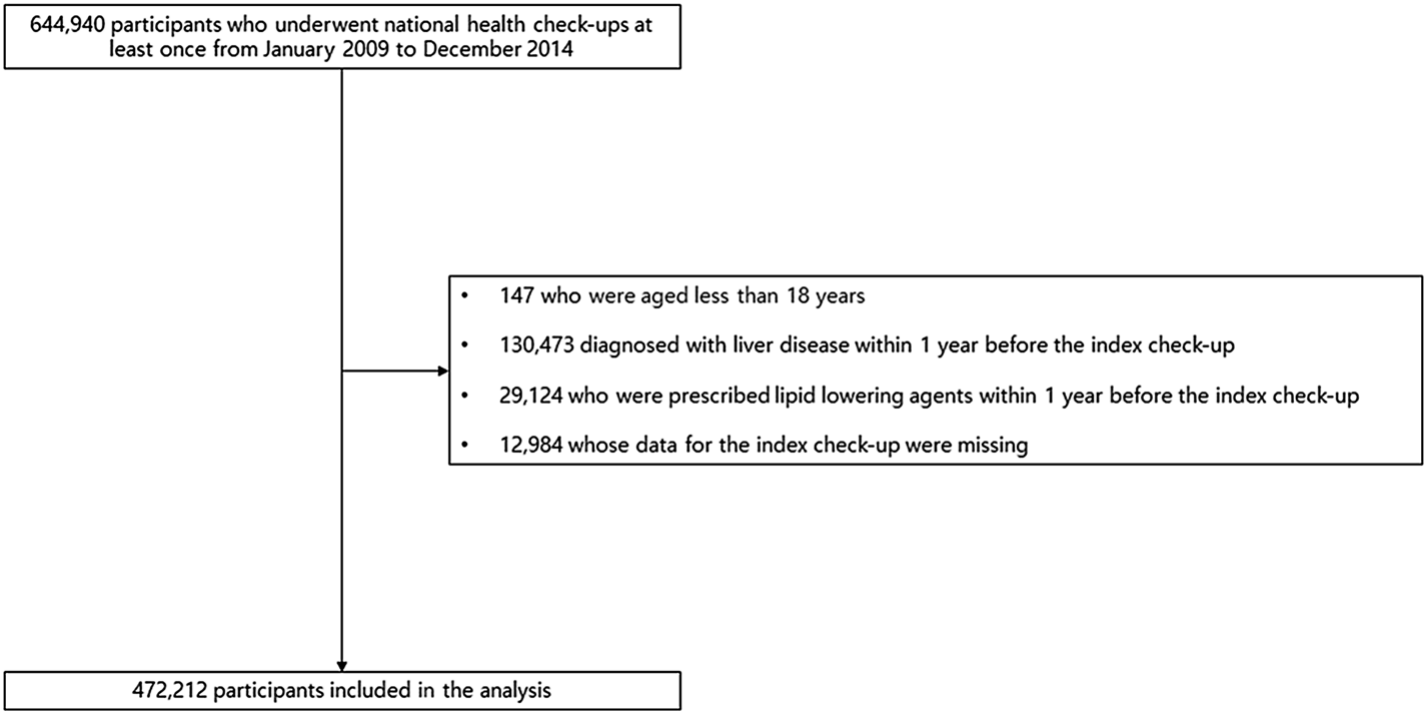
Overview of the study population.

A total of 644,940 participants underwent national health check-ups at least once from January 2009 to December 2014. Based on the prespecified exclusion criteria, the data of 472,212 participants were analyzed. Figure 1 shows a list of participants who were excluded. The study population was divided into four groups based on fatty liver index (FLI) quartile values (first quartile [Q1], 0–5.7; second quartile [Q2], 5.8–15.3; third quartile [Q3], 15.4–37.2; and fourth quartile [Q4], > 37.2). Table 1 summarizes the baseline characteristics of the study population according to FLI quartile values.

**Table 1:** Baseline characteristics.

	FLI quartile				p value
	Q1	Q2	Q3	Q4	
	(n = 118,045)	(n = 118,056)	(n = 118,055)	(n = 118,056)	
Age (year)	37.6 ± 13.0	44.3 ± 14.4	47.4 ± 14.3	46.2 ± 13.2	< 0.001
Male sex (%)	21,726 (18.4)	50,981 (43.2)	71,377 (60.5)	91,993 (77.9)	< 0.001
BMI (kg/m^2^)	20.2 ± 1.8	22.4 ± 1.9	24.3 ± 2.1	26.9 ± 3.1	< 0.001
Waist circumference (cm)	68.6 ± 5.1	76.2 ± 5.0	82.0 ± 5.2	89.4 ± 9.6	< 0.001
Systolic BP (mmHg)	112.6 ± 12.6	119.0 ± 13.8	123.6 ± 14.2	128.5 ± 14.9	< 0.001
Diastolic BP (mmHg)	70.4 ± 8.8	74.1 ± 9.4	76.9 ± 9.6	80.5 ± 10.3	< 0.001
Smoking					< 0.001
Non-smoker	97,806 (82.9)	80,299 (68.0)	65,684 (55.6)	46,602 (39.5)	
Ex-smoker	6143 (5.2)	11,693 (9.9)	17,550 (14.9)	22,262 (18.9)	
Current smoker	14,096 (11.9)	26,064 (22.1)	34,821 (29.5)	49,192 (41.7)	
Alcohol consumption (g/week)	34.1 ± 79.1	52.4 ± 109.4	74.4 ± 134.5	126.0 ± 184.1	< 0.001
Activity (MET-min/week)	359.1 ± 359.0	389.7 ± 392.0	391.8 ± 398.7	377.8 ± 384.3	< 0.001
Laboratory findings					
Fasting glucose (mg/dL)	89.1 ± 12.6	92.9 ± 16.8	97.0 ± 22.2	103.5 ± 29.8	< 0.001
Total cholesterol (mg/dL)	178.1 ± 33.2	189.7 ± 37.1	199.0 ± 38.8	209.7 ± 43.7	< 0.001
LDL cholesterol (mg/dL)	107.3 ± 210.6	115.5 ± 131.8	122.1 ± 114.3	121.7 ± 95.3	< 0.001
HDL cholesterol (mg/dL)	64.1 ± 22.7	58.8 ± 21.6	54.7 ± 25.7	51.3 ± 30.0	< 0.001
Triglyceride (mg/dL)	64.6 ± 24.0	93.0 ± 37.5	128.6 ± 58.4	210.1 ± 143.5	< 0.001
AST (U/L)	20.0 ± 9.0	22.1 ± 13.5	24.3 ± 17.9	31.0 ± 52.1	< 0.001
ALT (U/L)	14.6 ± 8.9	18.6 ± 15.6	23.8 ± 21.6	37.8 ± 51.6	< 0.001
AST/ALT ratio	1.5 ± 0.5	1.3 ± 0.7	1.1 ± 1.3	0.9 ± 0.5	< 0.001
GGT (U/L)	14.6 ± 6.0	20.4 ± 11.5	30.6 ± 23.5	67.2 ± 74.7	< 0.001
Creatinine (mg/dL)	0.9 ± 0.9	1.0 ± 1.2	1.0 ± 1.1	1.1 ± 1.2	< 0.001
Diabetes mellitus	2339 (2.0)	4748 (4.0)	7221 (6.1)	8258 (7.0)	< 0.001
Dyslipidemia	3040 (2.6)	4503 (3.8)	5405 (4.6)	5338 (4.5)	< 0.001
Hypertension	4181 (3.5)	10,771 (9.1)	17,460 (14.8)	20,892 (17.7)	< 0.001
Coronary artery disease	1271 (1.1)	2476 (2.1)	3391 (2.9)	3586 (3.0)	< 0.001
Arrhythmia	880 (0.7)	1152 (1.0)	1374 (1.2)	1188 (1.0)	< 0.001
Valvular heart disease	127 (0.1)	206 (0.2)	268 (0.2)	187 (0.2)	< 0.001
Peripheral artery disease	3205 (2.7)	5316 (4.5)	6932 (5.9)	6419 (5.4)	< 0.001
Cerebrovascular disease	1657 (1.4)	2994 (2.5)	3988 (3.4)	3631 (3.1)	< 0.001
Chronic obstructive pulmonary disease	26,419 (22.4)	26,872 (22.8)	27,164 (23.0)	25,399 (21.5)	< 0.001
Chronic kidney disease	110 (0.1)	234 (0.2)	234 (0.2)	258 (0.2)	< 0.001
Malignancy	1644 (1.4)	2149 (1.8)	2001 (1.7)	1430 (1.2)	< 0.001

Data are expressed as mean ± standard deviation or number (percent).

FLI, fatty liver index; BMI, body mass index; BP, blood pressure; MET, metabolic equivalent of task; LDL, low-density lipoprotein; HDL, high-density lipoprotein; AST, aspartate aminotransferase; ALT, alanine transaminase, GGT, gamma-glutamyltransferase.

An increase in the percentage of men, body mass index (BMI), proportion of current smokers, and amount of alcohol consumption was observed among participants from Q1 to Q4. Participants with higher FLI values had higher blood pressures, fasting glucose levels, and gamma-glutamyltransferase (GGT) levels and more comorbidities than those with lower FLI values. The lipid profiles became unfavorable with increasing FLI values.

The development of VTE was related to older age; more comorbidities; and higher BMI, glucose, cholesterol, aspartate aminotransferase (AST), and AST/alanine transaminase (ALT) ratio (Table 2). Alcohol consumption, ALT, and activity were not related to the development of VTE.

**Table 2:** Comparisons of baseline characteristics of participants.

	New-onset VTE (+) (n = 4499)	New-onset VTE (−) (n = 467,713)	p value
Age (year)	58.4 ± 14.6	43.8 ± 14.2	< 0.001
Male sex (%)	750 (47.4)	235,327 (50.0)	0.039
BMI (kg/m^2^)	24.1 ± 3.5	23.4 ± 3.4	< 0.001
Waist circumference (cm)	82.3 ± 9.27	79.1 ± 10.0	< 0.001
Systolic BP (mmHg)	125.9 ± 16.5	120.9 ± 15.1	< 0.001
Diastolic BP (mmHg)	77.4 ± 10.4	75.5 ± 10.2	< 0.001
Smoking			< 0.001
Non-smoker	1025 (64.8)	289,366 (61.5)	
Ex-smoker	220 (13.9)	57,428 (12.2)	
Current smoker	337 (21.3)	123,836 (26.3)	
Alcohol consumption (g/wk)	58.3 ± 153.7	71.8 ± 136.8	< 0.001
Activity (MET-min/wk)	362.1 ± 410.5	379.7 ± 383.9	0.09
Laboratory findings			
Fasting glucose (mg/dL)	99.3 ± 25.4	95.6 ± 22.0	< 0.001
Total cholesterol (mg/dL)	199.4 ± 39.0	194.1 ± 40.1	< 0.001
LDL cholesterol (mg/dL)	112.0 ± 57.6	116.6 ± 145.2	0.022
HDL cholesterol (mg/dL)	55.8 ± 32.4	57.2 ± 25.6	0.077
Triglyceride (mg/dL)	133.3 ± 86.7	124.1 ± 97.4	< 0.001
AST (U/L)	25.8 ± 15.6	24.3 ± 29.1	< 0.001
ALT (U/L)	23.7 ± 29.2	23.7 ± 30.7	0.994
AST/ALT ratio	1.3 ± 0.5	1.2 ± 0.8	< 0.001
GGT (U/L)	36.2 ± 56.3	33.2 ± 44.6	0.032
Creatinine (mg/dL)	1.0 ± 1.6	1.0 ± 1.1	0.363
Diabetes mellitus	209 (13.2)	22,357 (4.8)	< 0.001
Dyslipidemia	137 (8.7)	18,149 (3.9)	< 0.001
Hypertension	547 (34.6)	52,757 (11.2)	< 0.001
Coronary artery disease	130 (8.2)	10,594 (2.3)	< 0.001
Arrhythmia	54 (3.4)	4540 (1.0)	< 0.001
Valvular heart disease	10 (0.6)	778 (0.2)	< 0.001
Peripheral artery disease	230 (14.5)	21,642 (4.6)	< 0.001
Cerebrovascular disease	150 (9.5)	12,120 (2.6)	< 0.001
Chronic obstructive pulmonary disease	538 (34.0)	105,316 (22.4)	< 0.001
Chronic kidney disease	15 (0.9)	821 (0.2)	< 0.001
Malignancy	72 (4.6)	7152 (1.5)	< 0.001

Data are expressed as mean ± standard deviation or number (percent).

VTE, venous thromboembolism; BMI, body mass index; BP, blood pressure; MET, metabolic equivalent of task; LDL, low-density lipoprotein; HDL, high-density lipoprotein; AST, aspartate aminotransferase; ALT, alanine aminotransferase; GGT, γ-glutamyltransferase.

### Association between FLI and VTE incidence

**Figure 2:**
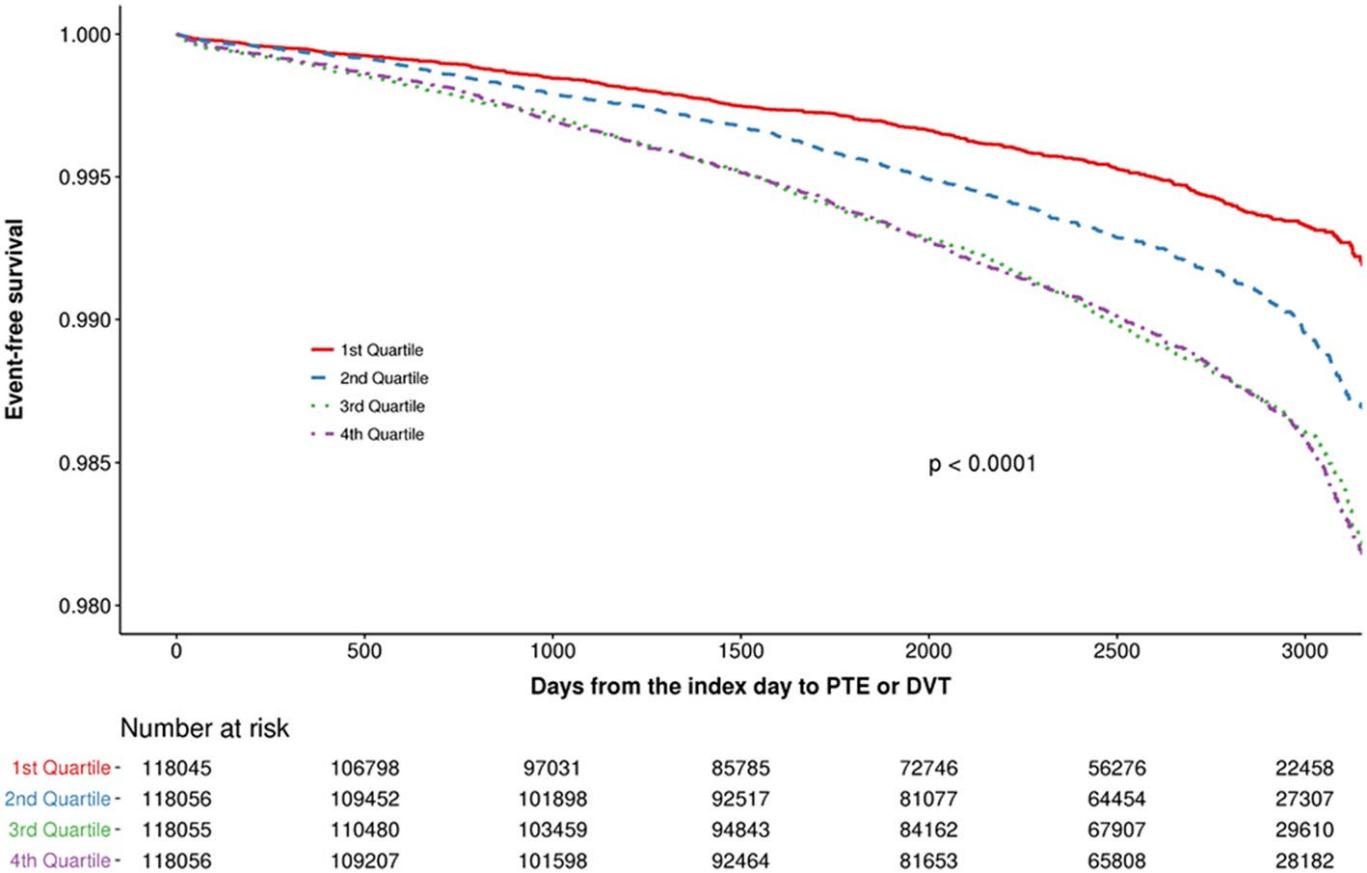
Cumulative incidence of new-onset venous thromboembolism according to their quartiles. PTE, pulmonary thromboembolism; DVT, deep vein thrombosis.

The median follow-up duration was 7.2 years (interquartile range, 4.6‒8.3), and new-onset VTE occurred in 4,499 individuals (1.0%). Figure 2 illustrates the cumulative incidence of new-onset VTE according to the FLI quartile groups. Supplementary Figs. 1 and 2 show the cumulative incidence of pulmonary thromboembolism (PTE) and deep vein thrombosis (DVT), respectively. The incidence of VTE tended to increase with increasing FLI values (Q1, 598 [0.5%]; Q2, 1033 [0.9%]; Q3, 1443 [1.2%]; and Q4, 1425 [1.2%]). Table 3 shows the univariate analysis results of new-onset VTE. In the age- and sex-adjusted multivariate model (model 1), the hazard ratio (HR) (95% confidence interval [CI]) was 1.47 (1.33‒1.62) for Q4 compared with Q1. When the model was further adjusted for clinical variables with *P* < 0.1 in the univariate analyses (model 2), the HR (95% CI) was 1.45 (1.30‒1.62) for Q4 compared with Q1 (Table 4).

**Table 3:** Univariate analysis of new-onset venous thromboembolism.

Characteristics	Value	Number	VTE (%)	HR	95% CI		p-value
					Lower	Upper	
Total	Total	472,212	4499 (1.0)	1.05	1.05	1.06	< 0.001
Age				1.05	1.05	1.06	< 0.001
Sex	Female	236,135	2392 (1.0)	Reference			
	Male	236,077	2107 (0.9)	0.83	0.78	0.09	< 0.001
BMI				1.06	1.05	1.07	< 0.001
Waist circumference				1.01	1.01	1.01	< 0.001
Systolic BP				1.02	1.01	1.02	< 0.001
Diastolic BP				1.02	1.01	1.02	< 0.001
Smoking	Non	290,391	2914 (1.0)	Reference			
	Ex	57,648	642 (1.1)	1.03	0.95	1.12	0.47
	current	124,173	943 (0.8)	0.77	0.72	0.83	< 0.001
Alcohol consumption				1.00	1.00	1.00	< 0.001
Activity				1.00	1.00	1.00	0.333
Laboratory findings							
Fasting glucose				1.01	1.00	1.01	< 0.001
Total cholesterol				1.00	1.00	1.00	< 0.001
LDL cholesterol				1.00	1.00	1.00	0.91
HDL cholesterol				1.00	1.00	1.00	< 0.001
Triglyceride				1.00	1.00	1.00	< 0.001
AST				1.00	1.00	1.00	0.009
ALT				1.00	1.00	1.00	0.513
GGT				1.00	1.00	1.00	0.003
Creatinine				1.00	0.98	1.02	0.95
Diabetes mellitus		449,646	3886 (0.9)				
		22,566	613 (2.7)	2.25	2.07	2.46	< 0.001
Dyslipidemia		453,926	4109 (0.9)				
		18,286	390 (2.1)	2.17	1.96	2.41	< 0.001
Hypertension		418,908	2965 (0.7)				
		53,304	1534 (2.9)	2.82	2.65	3.01	< 0.001
Coronary artery disease		461,488	4132 (0.9)				
		10,724	367 (3.4)	2.74	2.46	3.06	< 0.001
Arrhythmia		467,618	4346 (0.9)				
		4,594	153 (3.3)	2.90	2.46	3.41	< 0.001
Valvular heart disease		471,424	4466 (1.0)				
		788	33 (4.2)	3.29	2.33	4.63	< 0.001
Peripheral artery disease		450,340	3826 (0.9)				
		21,872	673 (3.1)	2.51	2.31	2.73	< 0.001
Cerebrovascular disease		459,942	4053 (0.9)				
		12,270	446 (3.6)	2.80	2.54	3.10	< 0.001
Chronic obstructive pulmonary disease		366,358	3011 (0.8)				
		105,854	1488 (1.4)	1.65	1.55	1.75	< 0.001
Chronic kidney disease		471,376	4463 (1.0)				
		836	36 (4.3)	3.57	2.57	4.96	< 0.001
Malignancy		464,988	4327 (0.9)				
		7224	172 (2.4)	2.01	1.73	2.35	< 0.001

VTE, venous thromboembolism; HR, hazard ratio; CI, confidential interval; BMI, body mass index; BP, blood pressure; LDL, low-density lipoprotein; HDL, high-density lipoprotein; AST, aspartate aminotransferase; ALT, alanine transaminase; GGT, gamma-glutamyltransferase.

**Table 4:** Association between fatty liver index and new-onset venous thromboembolism.

FLI quartile	Total (N)	Event (n, %)	Univariate			Model 1^a^			Model 2^b^		
			HR	95% CI	p-value	HR	95% CI	p-value	HR	95% CI	p-value
Q1	118,045	598 (0.5)	Reference			Reference			Reference		
Q2	118,056	1033 (0.9)	1.43	1.29–1.58	< 0.001	1.04	0.94‒1.15	0.456	1.04	0.94‒1.16	0.426
Q3	118,055	1443 (1.2)	1.89	1.72‒2.08	< 0.001	1.27	1.15‒1.40	< 0.001	1.27	1.14‒1.40	< 0.001
Q4	118,056	1425 (1.2)	1.94	1.77‒2.14	< 0.001	1.47	1.33‒1.62	< 0.001	1.45	1.30‒1.62	< 0.001

FLI, fatty liver index; HR, hazard ratio; CI, confidential interval.

^a^Cox proportional hazard models including age and sex as covariates.

^b^Cox proportional hazard models including age, sex, systolic blood pressure, diastolic blood pressure, smoking, alcohol consumption, fasting blood glucose, total cholesterol, low-density lipoprotein cholesterol, aspartate aminotransferase, diabetes mellitus, dyslipidemia, hypertension, coronary artery disease, arrhythmia, valvular heart disease, peripheral artery disease, cerebrovascular disease, chronic obstructive pulmonary disease, chronic kidney disease, and malignancy as covariates.

The same trend was observed when PTE and DVT were analyzed separately (Supplementary Tables 1 and 2).

Additionally, the analysis was performed using various FLI cutoff criteria as suggested in previous studies. The group with the highest FLI values had the highest risk for new-onset VTE regardless of the cutoff criteria (Supplementary Table 3).

### Subgroup analysis of clinical variables affecting VTE incidence

**Figure 3:**
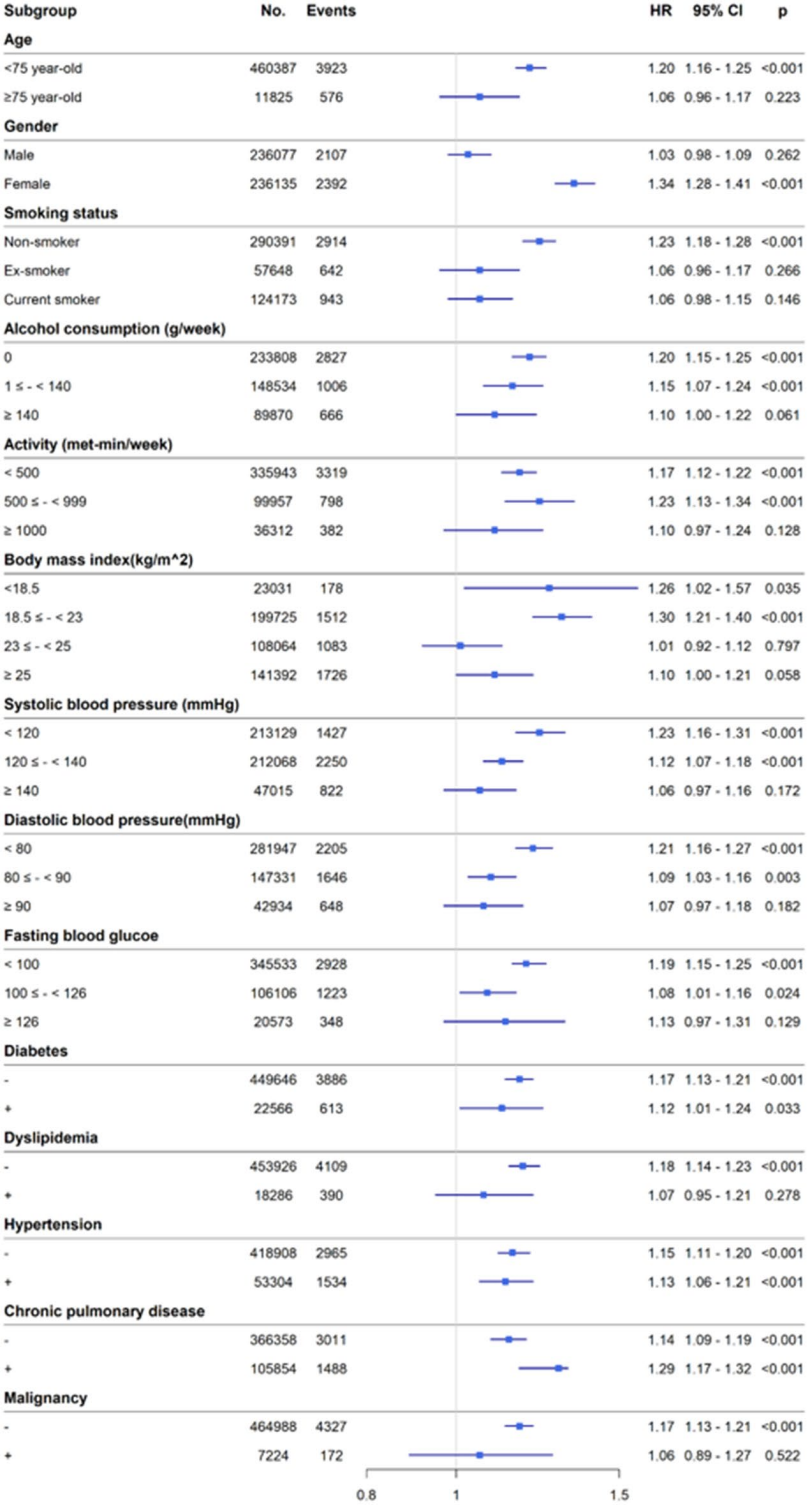
Forest plots of hazard ratios for new-onset venous thromboembolism stratified by various clinical characteristics. HR, hazard ratio; CI, confidence interval; FBS, fasting blood sugar; DM, diabetes mellitus; HTN, hypertension; COPD, chronic obstructive pulmonary disease.

Figure 3 shows the adjusted HRs according to subgroups. In all subgroups, the VTE incidence tended to increase with higher FLI values (Fig. 3).

## Discussion

This study investigated the relationship between NAFLD and VTE incidence in the Korean general population using national health check-up data. The results showed that FLI, a surrogate marker for fatty liver, was related to VTE risk, which was confirmed after adjusting for other clinical risk factors for VTE. This finding implies that individuals with higher FLI values and without overt liver disease have increased risk of VTE than those with lower FLI values. To the best of our knowledge, no previous study has investigated the relationship between FLI and VTE.

Despite the increasing prevalence of NAFLD, few studies have investigated the relationship between NAFLD and VTE incidence. In a case–control study of 138 consecutive patients with idiopathic VTE, Di Minno et al. found that the prevalence of NAFLD was 2.7-fold higher than that in the control group^13^. Furthermore, Stine et al. found that NASH etiology increased VTE risk by approximately 2.5-fold among hospitalized cirrhotic patients compared with other etiologies, implicating thrombosis mechanisms^14^. They also showed that liver transplant recipients with NASH were more likely to have portal vein thrombosis and VTE than those without^15^.

The development of VTE is related to venous stasis, endothelial dysfunction, and hypercoagulability. Among them, hypercoagulability and endothelial dysfunction could be implicated as the potential pathophysiologic mechanisms linking NAFLD to VTE^16^. Since the liver produces most factors associated with coagulation and fibrinolysis pathways, advanced liver disease of any etiology commonly exhibits disruptions in the coagulation pathway. NAFLD is associated with a relatively hypercoagulable state. Although the precise underlying mechanisms remain unknown, chronic liver inflammation is likely to be the most important trigger for disrupting the balance of the coagulation system^16–19^. Several studies found increased levels of individual procoagulant factors in patients with NAFLD. Kotronen et al. demonstrated increased activity of coagulation factors VIII, IX, XI, and XII in patients with NAFLD, independent of age, sex, and BMI^20^. In a study of histologically proven NAFLD, Verrijken et al. showed that NAFLD severity was independently associated with plasminogen activator inhibitor-1 levels among obese or overweight patients^21^. Tripodi et al. demonstrated a procoagulant imbalance in NAFLD using a new parameter called “thrombin generation,” a global coagulation procedure^22^. They found that thrombin generation increased sequentially from controls to steatosis or NASH and metabolic cirrhosis and that metabolic cirrhosis, the most advanced form, showed the highest factor VIII levels and lowest protein C levels.

This study had some limitations. First, FLI is a “surrogate marker” for fatty liver, not a diagnostic test. Although liver biopsy is considered the gold standard for diagnosing NAFLD, it is invasive, expensive, and has a possibility of sampling error. On the other hand, other imaging tests including abdominal ultrasound, computed tomography scan, and magnetic resonance imaging also have their limitations when used uniformly in a large number of patients. Many epidemiologic studies have used the FLI scoring system, which has shown relatively good diagnostic accuracy^23–25^. Second, NAFLD is defined as fat accumulation in the liver “without excessive alcohol intake” (< 140 g per week in women and 210 g per week in men). Thus, some individuals with alcoholic fatty liver might have been included in our cohort considering the amount of alcohol consumption. However, subgroup analysis showed equally significant results for nonalcoholics. Third, since this is an observational study, the causal relationship between NAFLD and increased risk of VTE could not be proven. Therefore, additional studies are needed to prove the causality.

In conclusion, FLI, a surrogate marker for NAFLD, was found to be associated with VTE incidence among the general adult population in Korea.

## Methods

### Ethical approval

This study was approved by the Institutional Review Board of Chungnam National University Hospital (No. 2023-04-001) and conducted in accordance with the Declaration of Helsinki (1989) by the World Medical Association. Informed consent was waived by the Institutional Review Board of Chungnam National University Hospital because anonymous clinical data were used in the analysis.

### Data source

This study analyzed data from the National Health Insurance Service-National Sample Cohort 2.0 (NHIS-NSC 2.0), a population-based sample cohort. The NHIS is a mandatory universal health insurance program that provides comprehensive medical care coverage to all residents in Korea, covering 97% of the total population. Approximately 2% of the total population in Korea was randomly sampled in 2006 with retrospective and prospective follow-up data from 2002 to 2015. The insured population (n = 48,222,537) was stratified into 2142 classes based on sex, age, region, eligibility status, and socioeconomic status. Subsequently, 2.1% of samples for each class were randomly selected (n = 1,021,208). Therefore, the NHIS-NCS 2.0 cohort can represent the whole Korean population^26^.

This cohort includes four databases: (1) beneficiary’s social demographic dataset; (2) dataset of medical claims with diagnoses based on the 10^th^ revision of the International Classification of Disease (ICD-10) codes, hospitalization, and treatment; (3) national health check-up dataset of cohort members; and (4) dataset of medical institutions.

Adults aged 18 years or older in Korea are recommended to undergo a national health screening every other year, consisting of a laboratory test, chest radiography, physical examination, and questionnaire regarding medical history and health-related behaviors (including alcohol consumption and smoking status). In the 2013 NHIS data, 72.1% of beneficiaries reported receiving a national health check-up (26). Mortality data, including the date and cause of death from the national statistical office’s death registration database, were also included.

The NHIS-NSC 2.0 data are available to any investigator with protocols approved by the NHIS review board. This study was approved by the Institutional Review Board of Chungnam National University Hospital, Daejeon, Korea.

### Study population

Adults over 18 years who had undergone national health check-ups more than once from 2009 to 2014 were enrolled in this study. The first national health check-up was defined as the index check-up, and the year of the index check-up was defined as the index year. The exclusion criteria were as follows: (a) individuals with liver disease; (b) those with prescribed medications, including lipid-lowering agents, within 2 years before the index check-up; and (c) those with missing data in the index check-up. The existence of each criterion was identified using two-year claims data before the index year.

### Definition of data

BMI was calculated by dividing body weight (kg) by height squared (m^2^). Smoking status was classified into non-smoker, ex-smoker, and current smoker. Alcohol consumption was estimated using standardized self-report questionnaires, including questions about how much alcohol was consumed at a time and how many days of the week alcohol was consumed. Subsequently, the amount of alcohol consumption was calculated by multiplying the answers from both questions. Physical activity was estimated using the following questions: how many days in a week do you perform 30 min of light exercise, 30 min of moderate exercise, and 20 min of vigorous exercise. Light exercise, moderate exercise, and vigorous exercise were defined as two metabolic equivalent of tasks (METs), three METs, and six METs, respectively. They were multiplied by 30, 30, and 20 min, respectively, and the respective number of days in a week and then summed.

The incidence of VTE was defined as the first occurrence of DVT or PTE, or death with a diagnosis of DVT or PTE. DVT and PTE were diagnosed based on ICD-10 codes (I80.1, I80.2, and I80.3 for DVT and I26 for PTE). Data were censored at the time of VTE occurrence, loss of NHIS eligibility because of death or immigration, or completion of study (December 31, 2015).

### Diagnosis of hepatic steatosis

FLI is widely known as a well-verified surrogate marker for identifying individuals with steatosis. It was calculated using four variables (triglycerides [TG], BMI, GGT, and waist circumference [WC]):${\text{FLI }} = \, {{\left( {{\text{e}}^{{0.{953 } \times {\text{loge}}\left( {{\text{TG}}} \right) \, + \, 0.{139} \times {\text{BMI }} + \, 0.{718} \times {\text{loge}}\left( {{\text{GGT}}} \right) \, + \, 0.0{53} \times {\text{WC }} - { 15}.{745}}} } \right)} \mathord{\left/ {\vphantom {{\left( {{\text{e}}^{{0.{953 } \times {\text{loge}}\left( {{\text{TG}}} \right) \, + \, 0.{139} \times {\text{BMI }} + \, 0.{718} \times {\text{loge}}\left( {{\text{GGT}}} \right) \, + \, 0.0{53} \times {\text{WC }} - { 15}.{745}}} } \right)} {\left( {{1 } + {\text{ e}}^{{0.{953} \times {\text{loge}}\left( {{\text{TG}}} \right) \, + \, 0.{139} \times {\text{BMI }} + \, 0.{718} \times {\text{loge}}\left( {{\text{GGT}}} \right) \, + \, 0.0{53} \times {\text{WC }} - { 15}.{745}}} } \right)}}} \right. \kern-0pt} {\left( {{1 } + {\text{ e}}^{{0.{953} \times {\text{loge}}\left( {{\text{TG}}} \right) \, + \, 0.{139} \times {\text{BMI }} + \, 0.{718} \times {\text{loge}}\left( {{\text{GGT}}} \right) \, + \, 0.0{53} \times {\text{WC }} - { 15}.{745}}} } \right)}} \times {1}00$.

Initially, an FLI value ≥ 60 was suggested as the criterion for diagnosing hepatic steatosis with a positive likelihood ratio of 4.3 in the general population^27^. Although the FLI is easy to obtain and calculated to screen fatty liver disease, there is insufficient evidence to support the diagnosis of fatty liver disease in Asians using FLI because they have lower BMI and WC compared with other ethnic populations^28^. Consequently, the study population was classified into quartiles according to their FLI value and then analyzed.

### Statistical analysis

Data are expressed as mean ± standard deviation for continuous variables and percentages for categorical variables. One-way analysis of variance and Pearson’s chi-square test were used to compare the difference between FLI quartiles. Kaplan‒Meier estimates and log-rank test were used to calculate and compare cumulative event rates between FLI quartile-based groups. Cox proportional hazards regression models were used to provide adjusted HRs with 95% CI for VTE incidence. Data were adjusted for age and sex (model 1). Moreover, clinical variables showing borderline association with new-onset VTE (*P* < 0.10) in univariate analyses were incorporated into model 2.

For subgroup analyses, FLI values were integrated into the statistical model as a continuous variable after log transformation. Statistical significance was considered at *P* < 0.05. R software (version 3.4.4) was used for statistical analyses (R Foundation for Statistical Computing, Vienna, Austria; www.r-project.org).

### Supplementary Information

Supplementary Figure 1.

Supplementary Figure 2.

Supplementary Table 1.

Supplementary Table 2.

Supplementary Table 3.

## Data Availability

The NHIS-NSC 2.0 is available to investigator with a protocol approved by the NHIS review board. The datasets is opened to investigator for a limited period of time. The analysis results of the current study are available from the corresponding author on reasonable request.
