# Supplementary figures and images for: Association of nonalcoholic fatty liver disease and venous thromboembolic disease in healthy adults in Korea: a nationwide study
Source: Sci Rep. 2023 Sep 26;13:16069. doi: 10.1038/s41598-023-42963-9 (PMC10522768; doi:10.1038/s41598-023-42963-9)

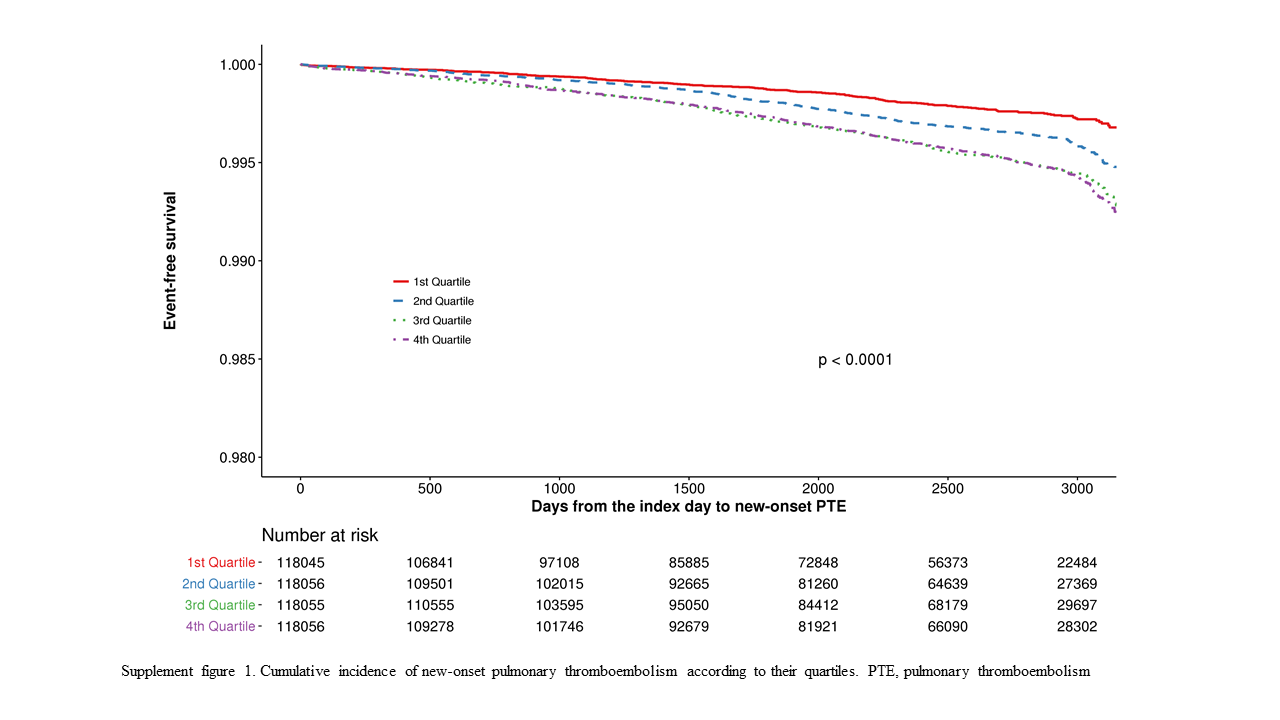

Supplement: Supplementary file 1 — Supplementary Figure 1. [file 41598_2023_42963_MOESM1_ESM.png]

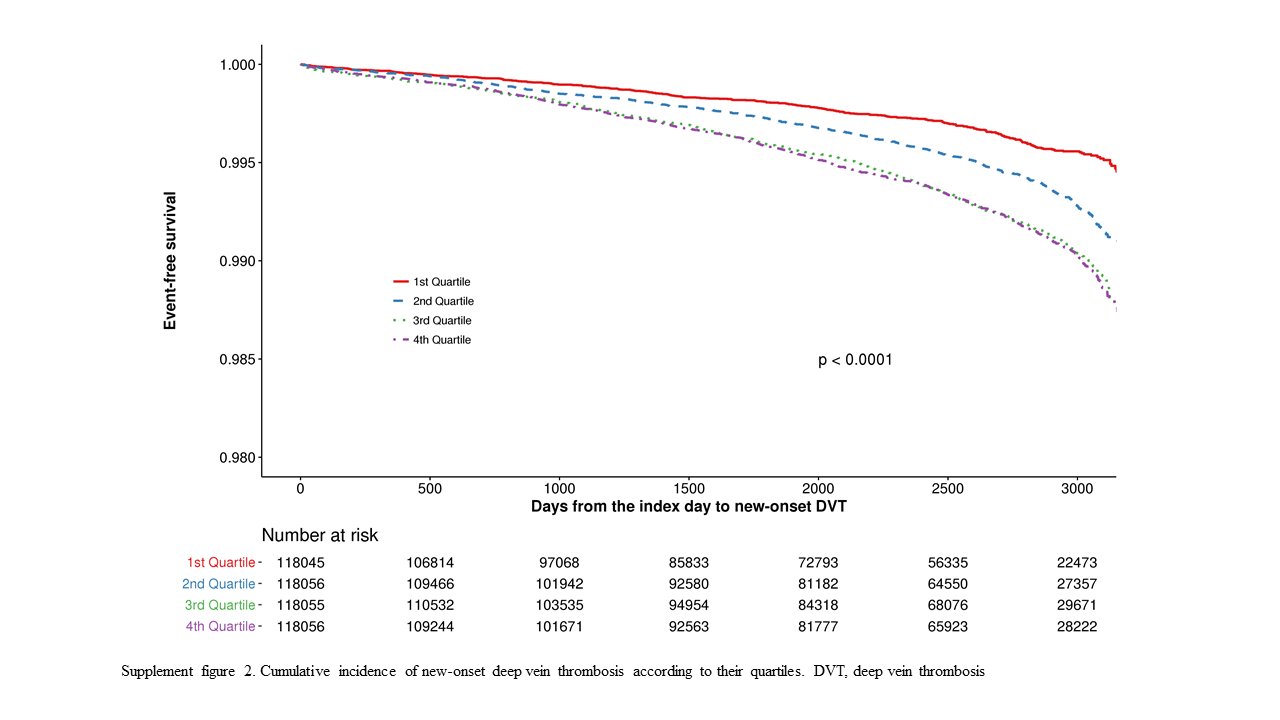

Supplement: Supplementary file 2 — Supplementary Figure 2. [file 41598_2023_42963_MOESM2_ESM.png]
