# Supplementary material for: Association of nonalcoholic fatty liver disease and venous thromboembolic disease in healthy adults in Korea: a nationwide study
Source: Sci Rep. 2023 Sep 26;13:16069. doi: 10.1038/s41598-023-42963-9 (PMC10522768; doi:10.1038/s41598-023-42963-9)
Supplement: Supplementary file 3 — Supplementary Table 1. [file 41598_2023_42963_MOESM3_ESM.docx]

Supplement table 1. Association between fatty liver index and new-onset pulmonary thromboembolism

| FLI quartile | Total (N) | Event (n, %) | Univariate | | | Model 1^a^ | | | Model 2^b^ | | |
| --- | --- | --- | --- | --- | --- | --- | --- | --- | --- | --- | --- |
|  |  |  | HR | 95% CI | p value | HR | 95% CI | p value | HR | 95% CI | p value |
| Q1 | 118,045 | 248 (0.2) | Reference | | | Reference | | | Reference | | |
| Q2 | 118,056 | 460 (0.3) | 1.37 | 1.17-1.61 | <0.001 | 0.90 | 0.77-1.06 | 0.192 | 0.90 | 0.76-1.06 | 0.189 |
| Q3 | 118,055 | 584 (0.5) | 1.89 | 1.63-2.19 | <0.001 | 1.10 | 0.95-1.28 | 0.208 | 1.09 | 0.93-1.27 | 0.305 |
| Q4 | 118,056 | 591 (0.5) | 1.97 | 1.69-2.28 | <0.001 | 1.31 | 1.12-1.53 | 0.001 | 1.29 | 1.09-1.52 | 0.004 |

FLI = fatty liver index; HR = hazard ratio; CI = confidential interval

^a^Cox proportional hazard models including age and gender as covariates

^b^Cox proportional hazard models including age, gender, systolic blood pressure, diastolic blood pressure, smoking, alcohol consumption, fasting blood glucose, total cholesterol, low-density lipoprotein cholesterol, aspartate aminotransferase, diabetes mellitus, dyslipidemia, hypertension, coronary artery disease, arrhythmia, valvular heart disease, peripheral artery disease, cerebrovascular disease, chronic obstructive pulmonary disease, chronic kidney disease, and malignancy as covariates
